# Supplementary figures and images for: Fibril-induced glutamine-/asparagine-rich prions recruit stress granule proteins in mammalian cells
Source: Life Sci Alliance. 2019 Jul 2;2(4):e201800280. doi: 10.26508/lsa.201800280 (PMC6607448; doi:10.26508/lsa.201800280)

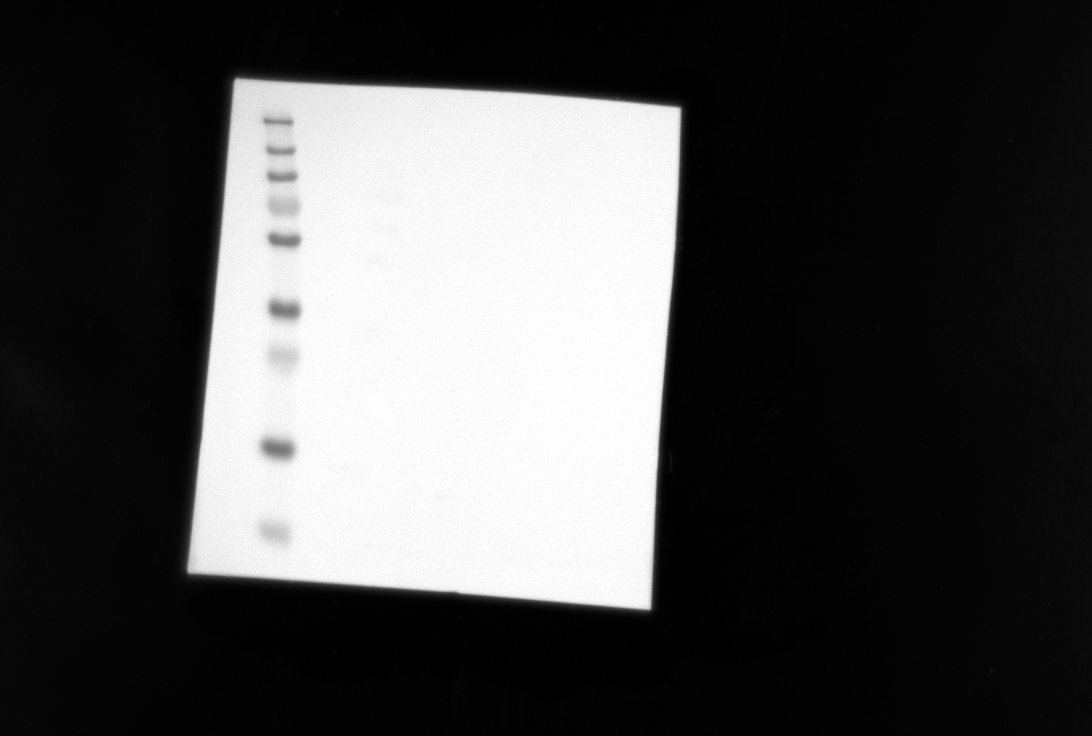

Supplement: Supplementary file 2 [file LSA-2018-00280_SdataF5A.tif]

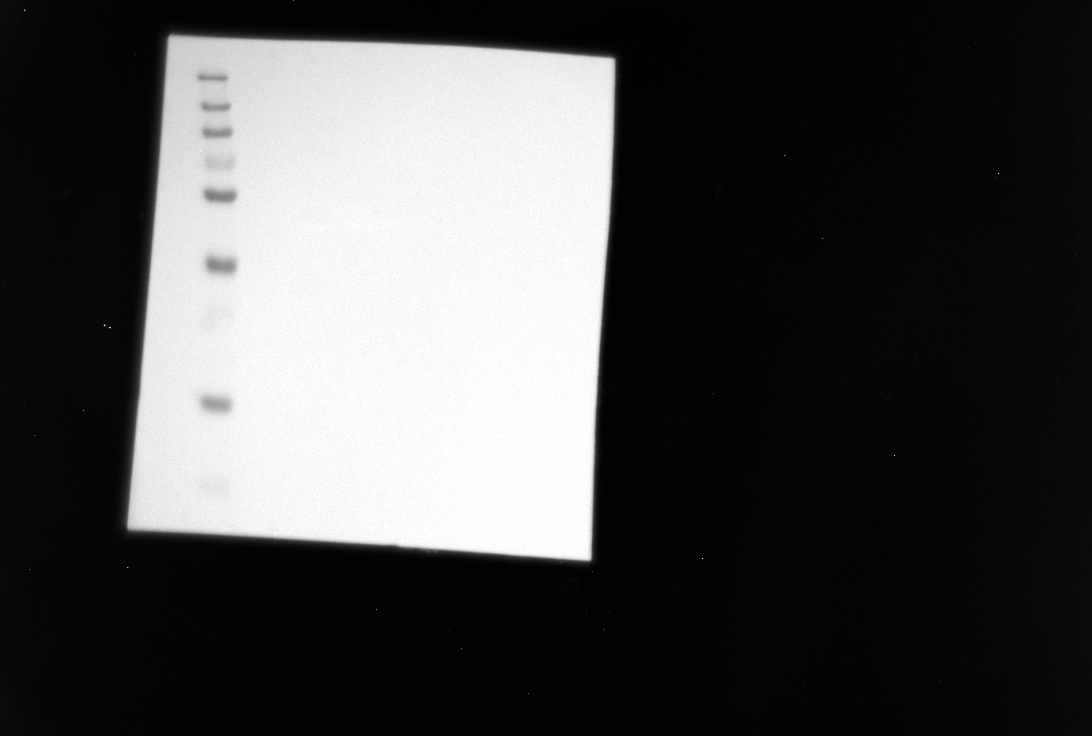

Supplement: Supplementary file 3 [file LSA-2018-00280_SdataF5B.tif]

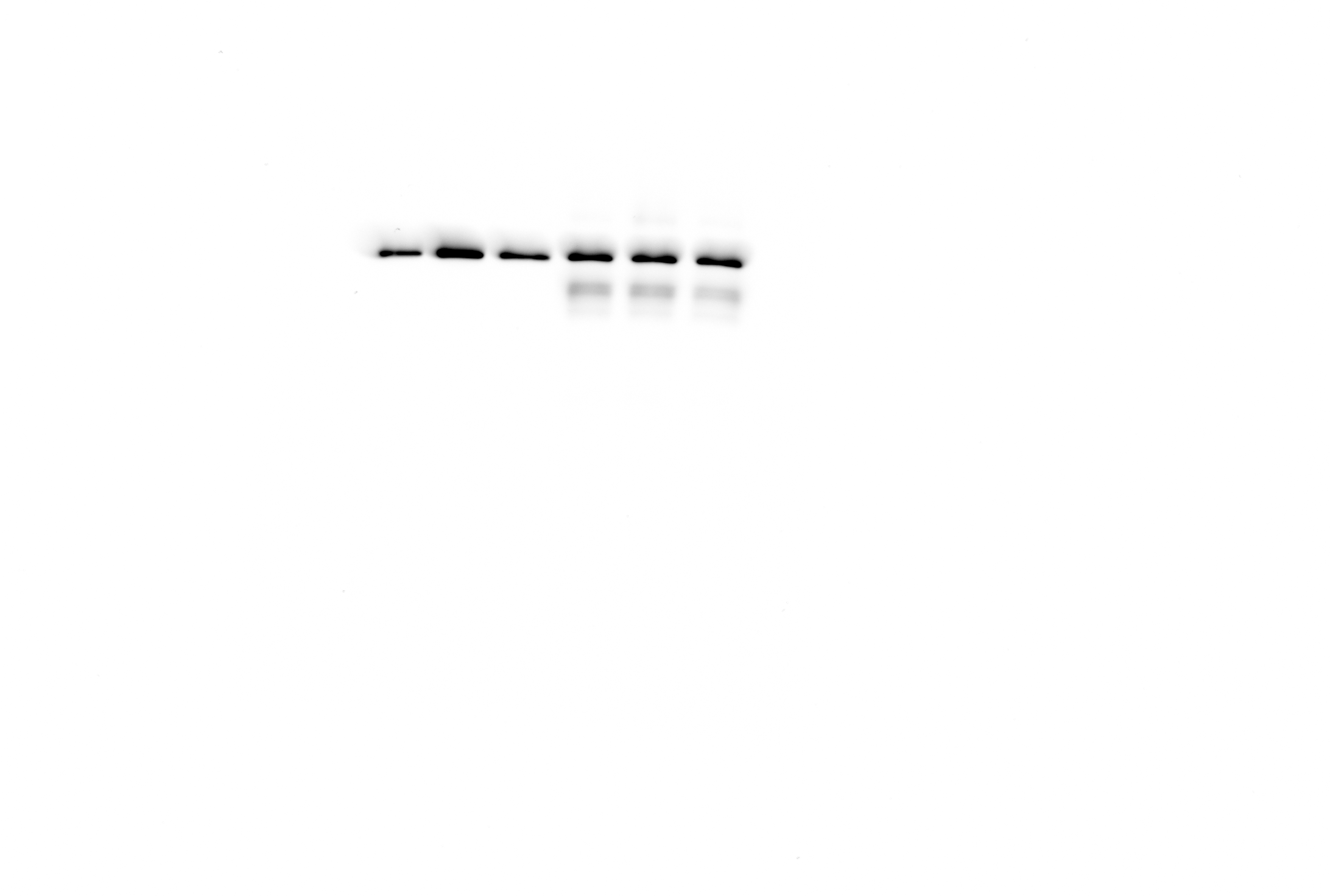

Supplement: Supplementary file 4 [file LSA-2018-00280_SdataF5C.tif]

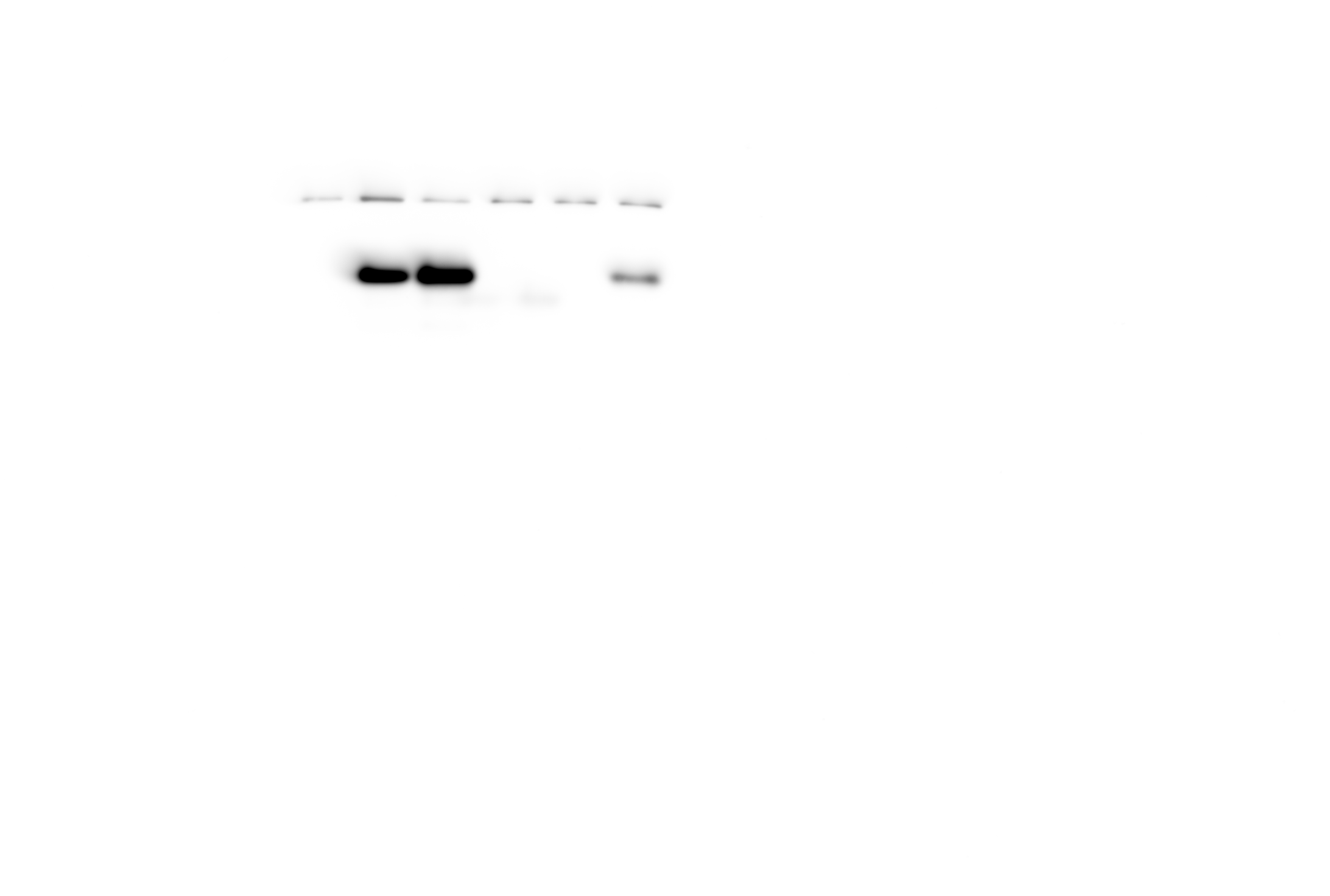

Supplement: Supplementary file 5 [file LSA-2018-00280_SdataF5D.tif]

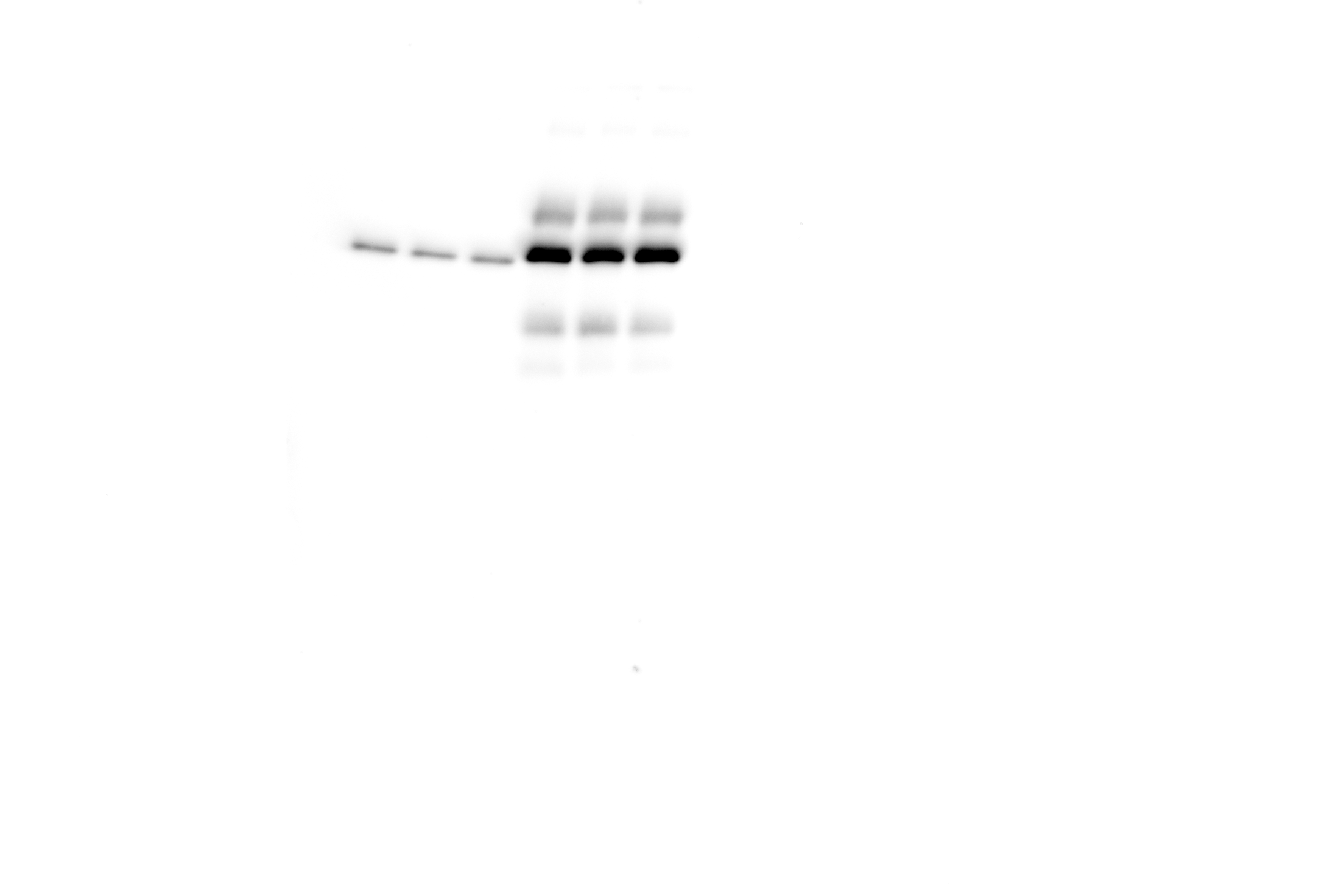

Supplement: Supplementary file 6 [file LSA-2018-00280_SdataF5E.tif]

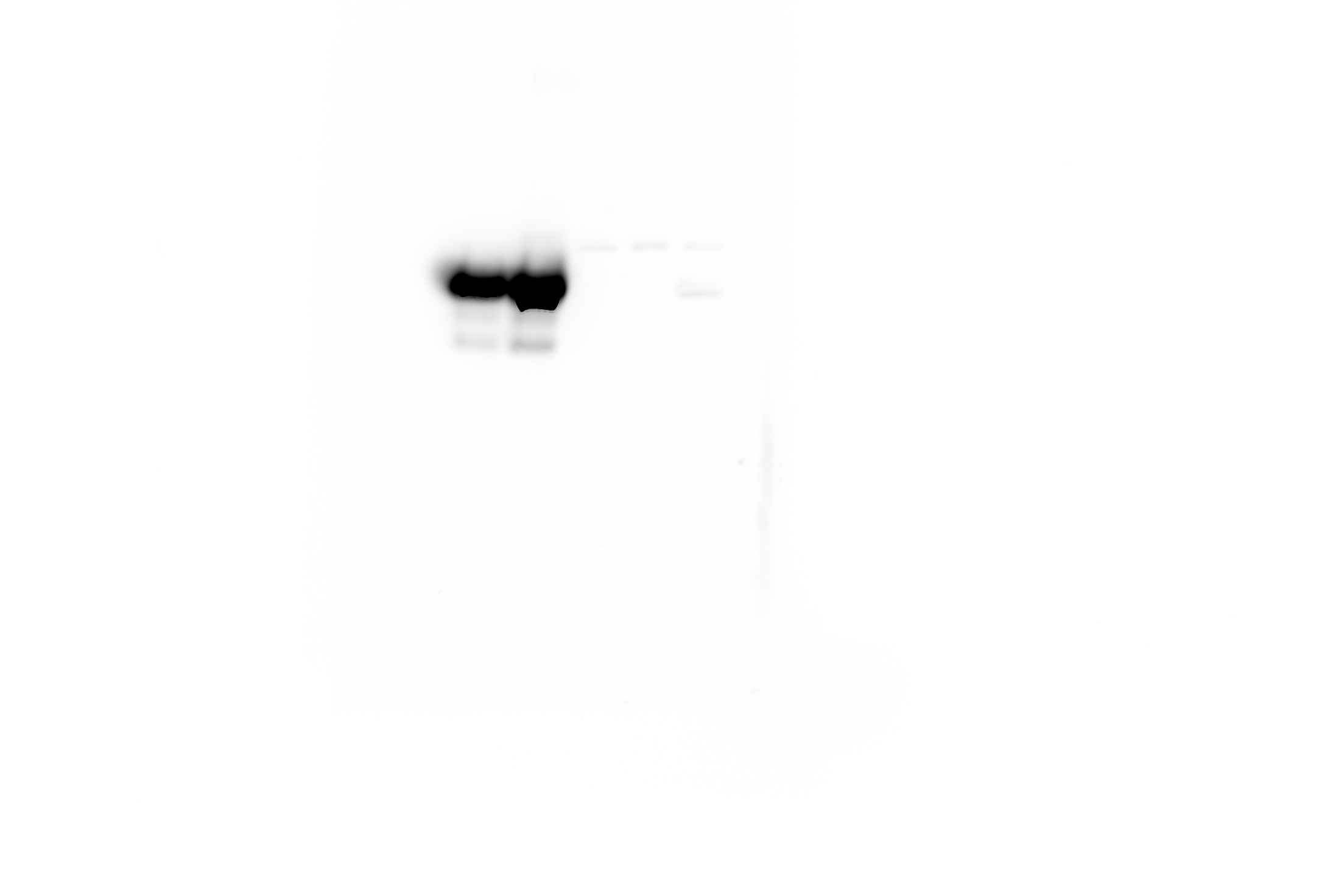

Supplement: Supplementary file 7 [file LSA-2018-00280_SdataF5F.tif]

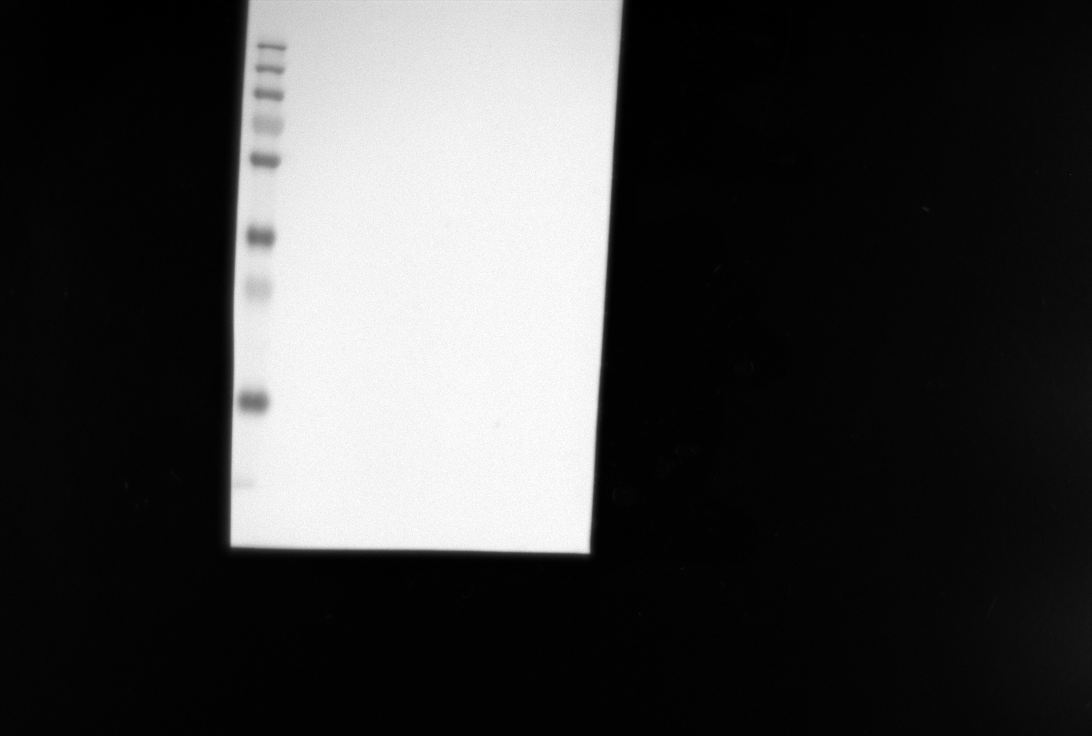

Supplement: Supplementary file 8 [file LSA-2018-00280_SdataF5G.tif]

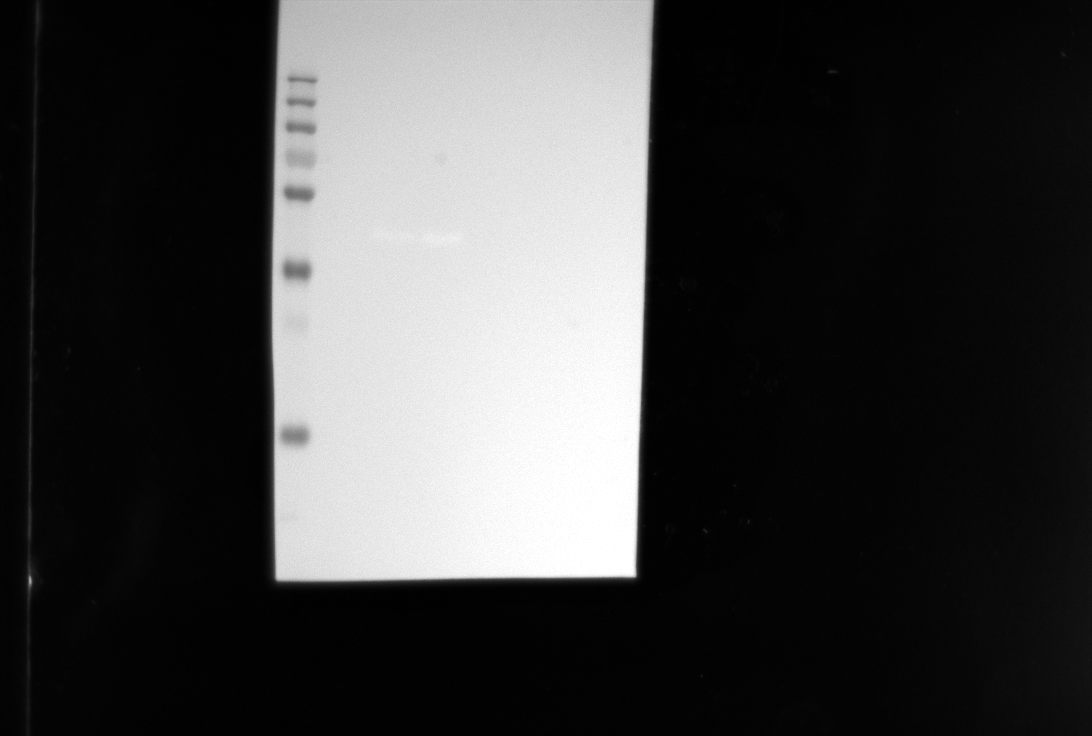

Supplement: Supplementary file 9 [file LSA-2018-00280_SdataF5H.tif]

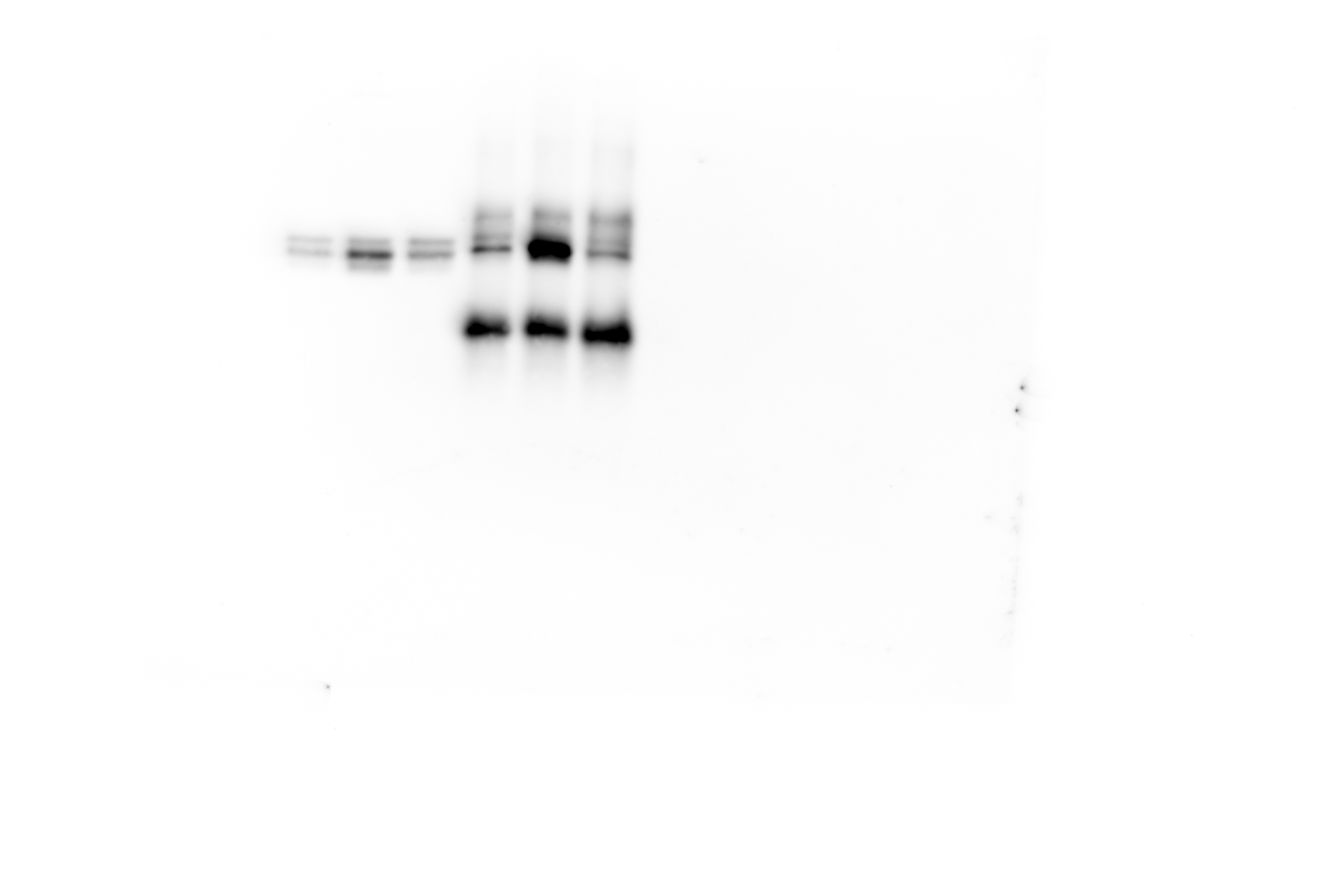

Supplement: Supplementary file 10 [file LSA-2018-00280_SdataFS2A.tif]

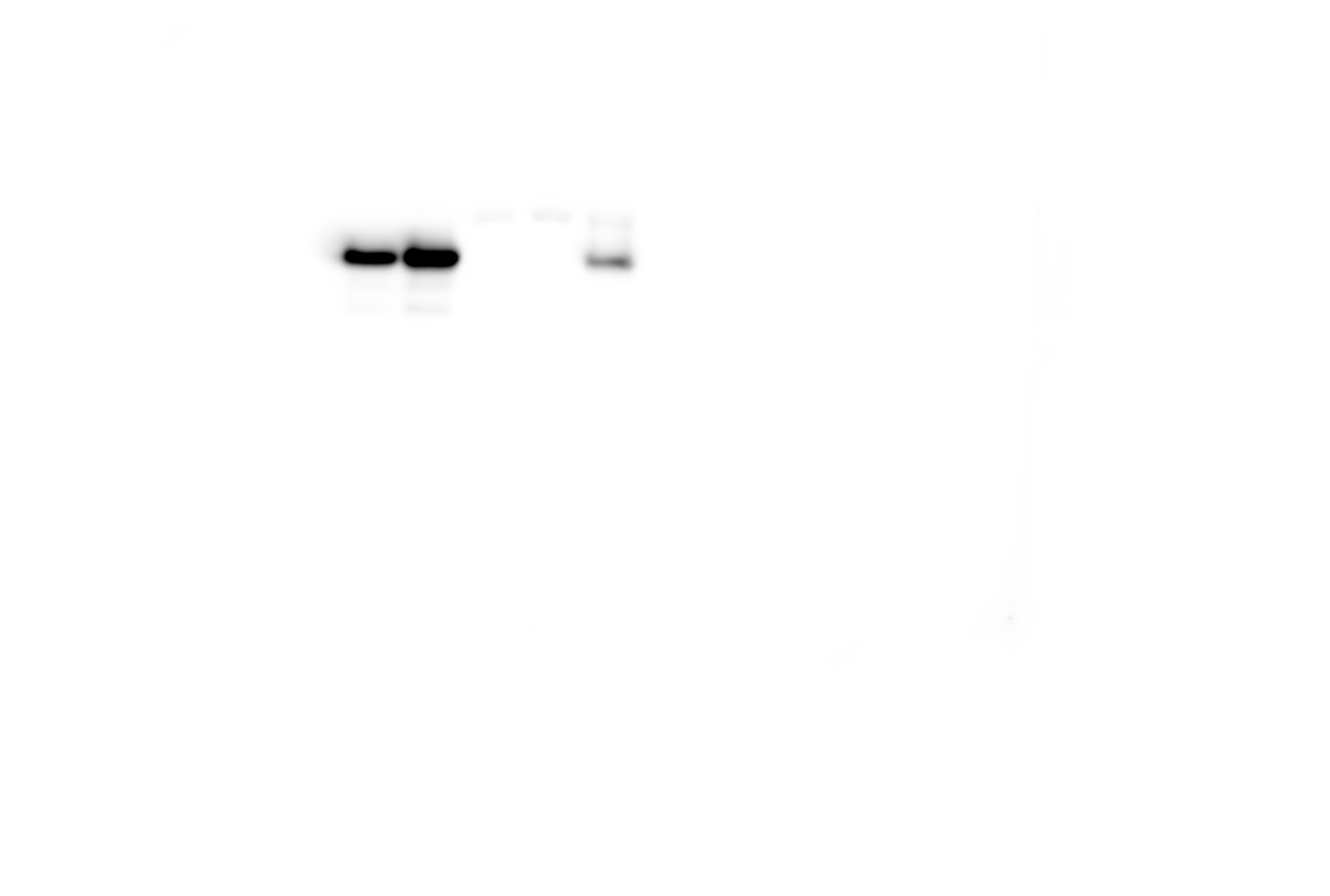

Supplement: Supplementary file 11 [file LSA-2018-00280_SdataFS2B.tif]

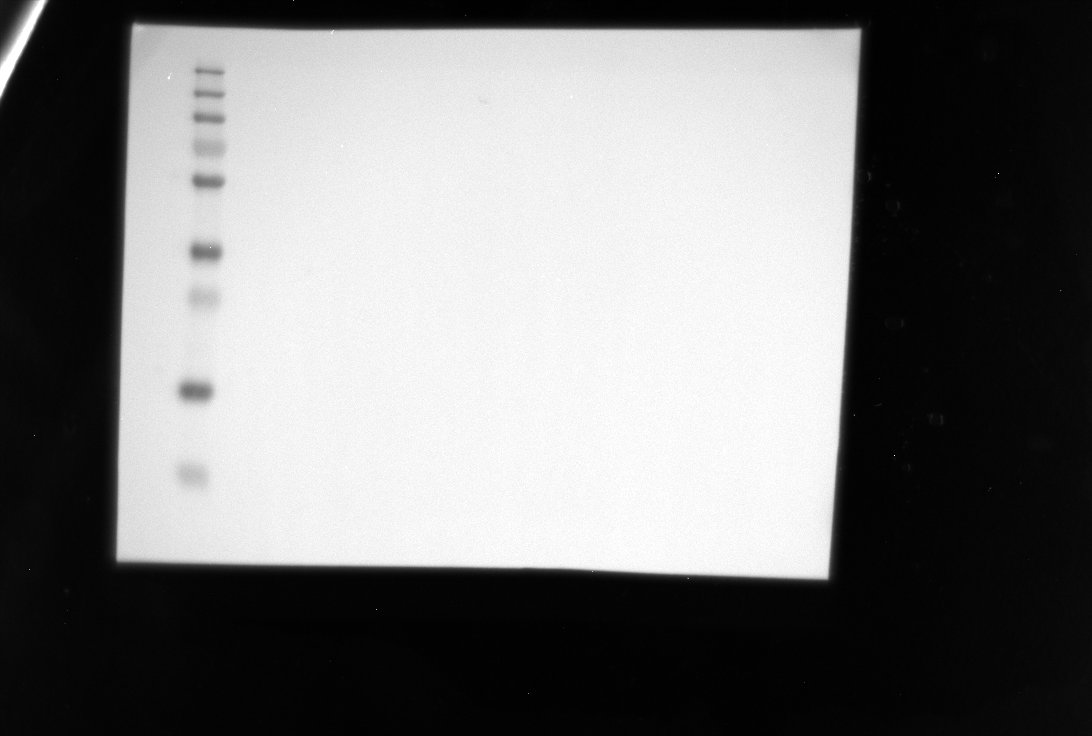

Supplement: Supplementary file 12 [file LSA-2018-00280_SdataFS2C.tif]

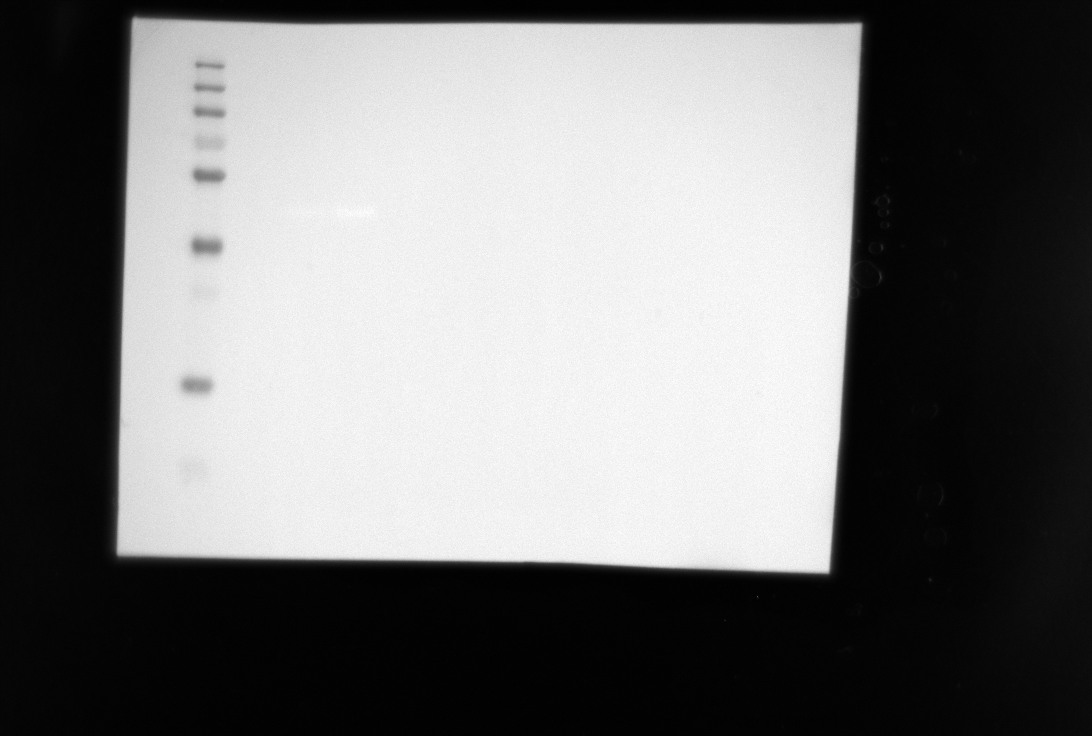

Supplement: Supplementary file 13 [file LSA-2018-00280_SdataFS2D.tif]
